# A Lipidomic Signature Complements Stemness Features Acquisition in Liver Cancer Cells

**DOI:** 10.3390/ijms21228452

**Published:** 2020-11-10

**Authors:** Irma Magaly Rivas Serna, Ilaria Romito, Andrea Maugeri, Oriana Lo Re, Sebastiano Giallongo, Gianluigi Mazzoccoli, Jude A. Oben, Giovanni Li Volti, Tommaso Mazza, Anna Alisi, Manlio Vinciguerra

**Affiliations:** 1 International Clinical Research Center, St. Anne’s University Hospital, 65691 Brno, Czech Republic; irma.magaly.rivas.serna@fnusa.cz (I.M.R.S.); oriana.lore@fnusa.cz (O.L.R.); sebastiano.giallongo@fnusa.cz (S.G.); 2 Research Area for Multifactorial Diseases, Research Unit of Molecular Genetics of Complex Phenotypes, Bambino Gesù Children’s Hospital, IRCCS, 00165 Rome, Italy; ilariaromito@gmail.com; 3 Department of Medical and Surgical Sciences and Advanced Technologies “GF Ingrassia”, University of Catania, 95123 Catania, Italy; andreamaugeri88@gmail.com; 4 Department of Biology, Faculty of Medicine, Masaryk University, 62500 Brno, Czech Republic; 5 Department of Medical Sciences and Chronobiology Laboratory, Fondazione IRCCS Casa Sollievo della Sofferenza, 71013 San Giovanni Rotondo, Italy; g.mazzoccoli@operapadrepio.it; 6 Institute for Liver and Digestive Health, Division of Medicine, University College London (UCL), London NW32PF, UK; j.oben@ucl.ac.uk; 7 Department of Biomedical and Biotechnological Sciences, University of Catania, 95131 Catania, Italy; livolti@unict.it; 8 Bioinformatics Unit, Fondazione IRCCS Casa Sollievo della Sofferenza, 71013 San Giovanni Rotondo, Italy; t.mazza@css-mendel.it; 9 ERA Chair in Translational Stem Cell Biology, Medical University of Varna, 9002 Varna, Bulgaria

**Keywords:** macroH2A1, FAK, HCC, stemness, cancer stem cells

## Abstract

Lipid catabolism and anabolism changes play a role in stemness acquisition by cancer cells, and cancer stem cells (CSCs) are particularly dependent on the activity of the enzymes involved in these processes. Lipidomic changes could play a role in CSCs’ ability to cause disease relapse and chemoresistance. The exploration of lipid composition and metabolism changes in CSCs in the context of hepatocellular cancer (HCC) is still incomplete and their lipidomic scenario continues to be elusive. We aimed to evaluate through high-throughput mass spectrometry (MS)-based lipidomics the levels of the members of the six major classes of sphingolipids and phospholipids in two HCC cell lines (HepG2 and Huh-7) silenced for the expression of histone variant macroH2A1 (favoring stemness acquisition), or silenced for the expression of focal adhesion tyrosine kinase (FAK) (hindering aggressiveness and stemness). Transcriptomic changes were evaluated by RNA sequencing as well. We found definite lipidomic and transcriptomic changes in the HCC lines upon knockdown (KD) of macroH2A1 or FAK, in line with the acquisition or loss of stemness features. In particular, macroH2A1 KD increased total sphingomyelin (SM) levels and decreased total lysophosphatidylcholine (LPC) levels, while FAK KD decreased total phosphatidylcholine (PC) levels. In conclusion, in HCC cell lines knocked down for specific signaling/epigenetic processes driving opposite stemness potential, we defined a lipidomic signature that hallmarks hepatic CSCs to be exploited for therapeutic strategies.

## 1. Introduction

Hepatocellular carcinoma (HCC) is the second leading cause of cancer-related death in the world [1]. In advanced HCC, the liver maintains only a poor level of function, which limits patients’ eligibility for surgery. Like other solid tumors, HCC contains a population of cancer stem cells (CSCs), which are associated with metastatic potential and disease relapse [2]. Hepatic CSCs are able to self-renew and differentiate to give rise to virtually any HCC cell type [2]. Remarkably, while CSCs themselves do not rapidly proliferate, their progeny have a high proliferative capacity and augment the tumor mass. Sorafenib, the gold standard chemotherapeutic agent for HCC patients, increases the median survival time by only a few months [3]. In fact, although chemotherapeutic drugs suppress the proliferating cells that comprise the main tumor mass [4], CSCs are very resilient to chemotherapy. It is thus necessary to develop therapies that specifically target CSCs to enhance patients’ survival and quality of life. Although several CSC markers have been identified, including CD133, CD90, CD44, oval cell marker OV6, EpCAM, CD13, CD24, DLK1, α2δ1, ICAM-1, CD47, Lgr5, and keratin19 [5], these cells possess a complex cell physiology and pathophysiological role, with considerable crosstalk and redundancy in signaling pathways.

Mass spectrometry (MS) approaches in liver cancer compared to the matched nontumorous tissue revealed that the saturation levels of triacylglycerol (TAG), and the levels of phosphatidylcholine (PC), phosphatidylethanolamine (PE) and phosphatidylinositol (PI), sphingomyelin (SM), and ceramide (Cer) provided lipid marker signatures of HCC progression [6,7,8]. Accordingly, several basic and preclinical reports suggested that supplementation with unsaturated fatty acids (FA) and TAG aggravated HCC growth, while saturated ones impaired it [8,9,10,11,12]; these effects are believed to be due to alterations of membrane fluidity and glucose metabolism. In this respect, it has been recently shown that HCC cells can exploit an alternative fatty acid desaturation pathway, which desaturates palmitate to the unusual fatty acid sapienate to support membrane biosynthesis during proliferation [13]. The research into the disorder of lipid composition and metabolism in CSCs is more limited, but might provide new insights into HCC mechanisms of relapse and refractoriness to therapy. Both lipid catabolism and anabolism alterations seem to contribute to stemness acquisition in CSCs, including lipid uptake, de novo lipogenesis, lipid desaturation, lipolysis, lipophagy, and fatty acid oxidation [14]. Accordingly, it appears that CSCs are extremely reliant on the activity of the enzymes involved in these processes, such as stearoyl-CoA desaturase 1 (SCD1) and 3-hydroxy-3-methylglutharyl-coenzyme A reductase (HMG-CoAR) [15]. In addition, increase in extracellular lipid uptake contributes to lipid droplets (LDs) accumulation and tumor initiation capacity in CSCs [16], a phenomenon that we have recently described for the first time in liver CSCs [17,18,19]. Whereas therapeutic strategies to eradicate nonhepatic CSCs based on their aberrant lipid metabolism have been envisaged [20], a lipid marker signature of liver CSCs obtained through high-throughput mass spectrometry (MS)-based lipidomics remains elusive, and it could guide novel therapeutic approaches. In the present study, we characterize the levels of the members of the six major classes of sphingolipids and phospholipids [Cer, PC, lysophosphatidylcholine (LPC), PE, lysophosphatidylethanolamine (LPE), SM] in two HCC cell lines: HepG2 and Huh-7 were either silenced for the expression of histone variant macroH2A1, which contributes to transform them in liver CSC-like cells [17,18,19], or silenced for the expression of focal adhesion tyrosine kinase (FAK), which in turn is capable of inhibiting their aggressiveness and stemness [21,22].

## 2. Results

### 2.1. Transcriptomic Changes in HepG2 and Huh-7 Cells, upon Knockdown (KD) of macroH2A1 (CSC-like) or KD of FAK (CSC Inhibition)

HepG2 and Huh-7 are widely used HCC cell lines used for research in hepatology, and they display large variations in their genetic and metabolic makeups. We recently discovered that shRNA-mediated silencing of the large histone variant macroH2A1 induces similar profound signaling, metabolic and secretomic changes in both cell lines, transforming them in CSC-like cells, which proliferate slowly, have a high tumorigenic potential, rely on glycolytic pathways, are resistant to hypoxic environment, accumulate LDs, express reprogramming genes, and evade the adaptive immune system [17,18,19] (Supplemental Figure S1). Conversely, shRNA-mediated silencing of FAK sets off opposite phenotypes [21] (Supplemental Figure S1). With respect to the transcriptomic changes accompanying the dedifferentiation occurring during the malignant process, here, we employed previously obtained RNA-Seq profiles [17,18,19] and novel data obtained by PCR Open Array analysis, upon macroH2A1 or FAK KD, respectively.

**Figure 1:**
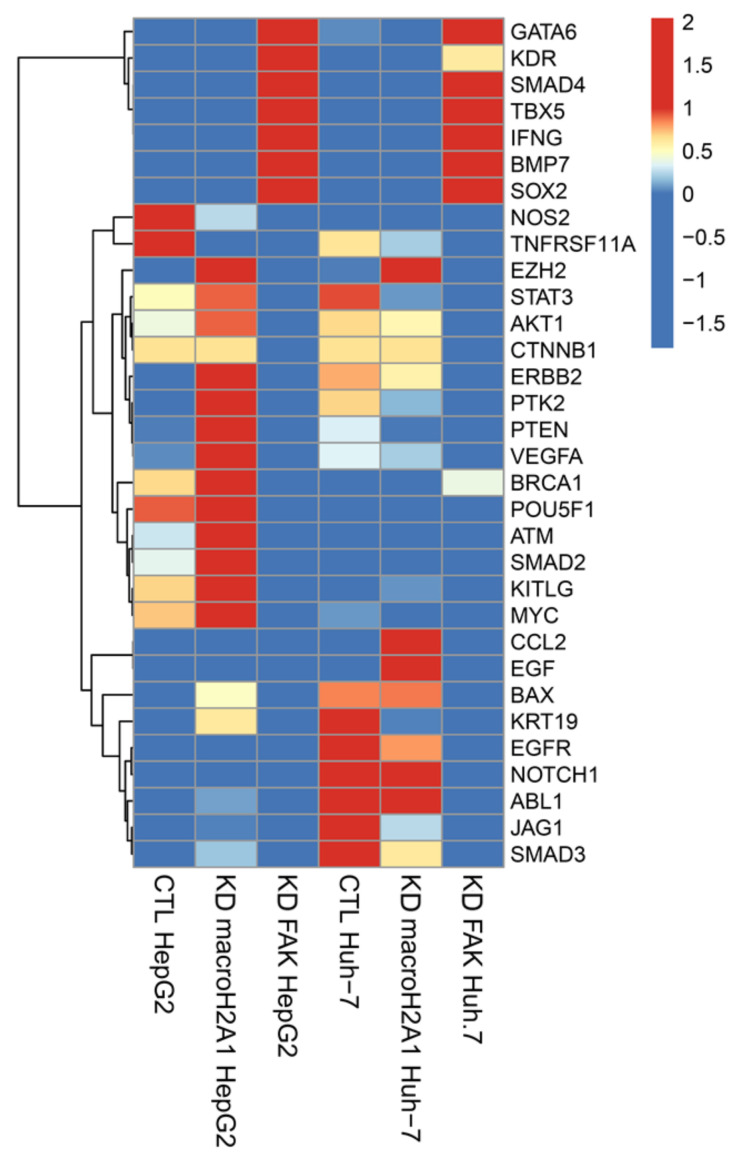
Differentially expressed genes related to reprogramming and cancer stemness in control (CTL), macroH2A1 knockdown (KD), and FAK KD cells (Huh-7 and HepG2). Heatmap of standardized counts per million (cpm) values of reprogramming and cancer stemness-related genes in macroH2A1KD versus CTL and KAD KD versus CTL, in Huh-7 cells (left) and in HepG2 cells (right).

Comparing the results from the two sets of data, we extrapolated the expression of 32 key genes involved in reprogramming and cancer stemness acquisition (*GATA6*, *KDR*, *SMAD4*, *TBX5*, *IFNG*, *BMP7*, *SOX2*, *NOS2*, *TNFRSF11A*, *EZH2*, *STAT3*, *AKT1*, *CTNNB1*, *ERBB2*, *PTK2*, *PTEN*, *VEGFA*, *BRCA1*, *POU5F1*, *ATM*, *SMAD2*, *KITLG*, *MYC*, *CCL2*, *EGF*, *BAX*, *KRT19*, *EGFR*, *NOTCH1*, *ABL1*, *JAG1*, and *SMAD3*) (Figure 1).

We found that macroH2A1 KD HepG2 cells, but not FAK KD HepG2 cells, displayed significant increase (*p* < 0.01) in the mRNA levels of master reprogramming transcription factors Oct-4 (POUF51) and MYC, as well as in many oncogenes (*EZH2*, *STAT3*, *AKT1*, *ERBB2*, *PTK2*, *VEGFA*, *ATM*, *KITLG*), compared to control cells (Figure 1) [18]. These differences were less marked or absent in Huh-7 cells (Figure 1). Moreover, we found that FAK KD HepG2 and Huh-7 cells, but not macroH2A1 KD HepG2 and Huh-7 cells, displayed a simultaneous significant increase (*p* < 0.01) in the mRNA levels of cancer stemness suppressor genes *GATA6*, *SMAD4*, and *BMP7*, and of tumor-promoting genes *KDR* and *SOX2* (Figure 1). Finally, compared to macroH2A1 KD Huh-7 cells, FAK KD cells exhibited a significant reduction (*p* < 0.01) in the mRNA levels of tumor-promoting genes *CCL2*, *EGF*, *BAX*, *KRT19*, *EGFR*, *NOTCH1*, *ABL1*, and *SMAD3* (Figure 1). Overall, these complex transcriptomic changes suggest that KD of macroH2A1 or of FAK triggers fairly polarized phenotypes, CSC-like (macroH2A1 KD) and anti-CSC (FAK KD), respectively, consistent with previous reports [17,18,19,21].

### 2.2. Lipid Class Composition in Huh-7 and HepG2 Cells Depleted for macroH2A1 or FAK

**Figure 2:**
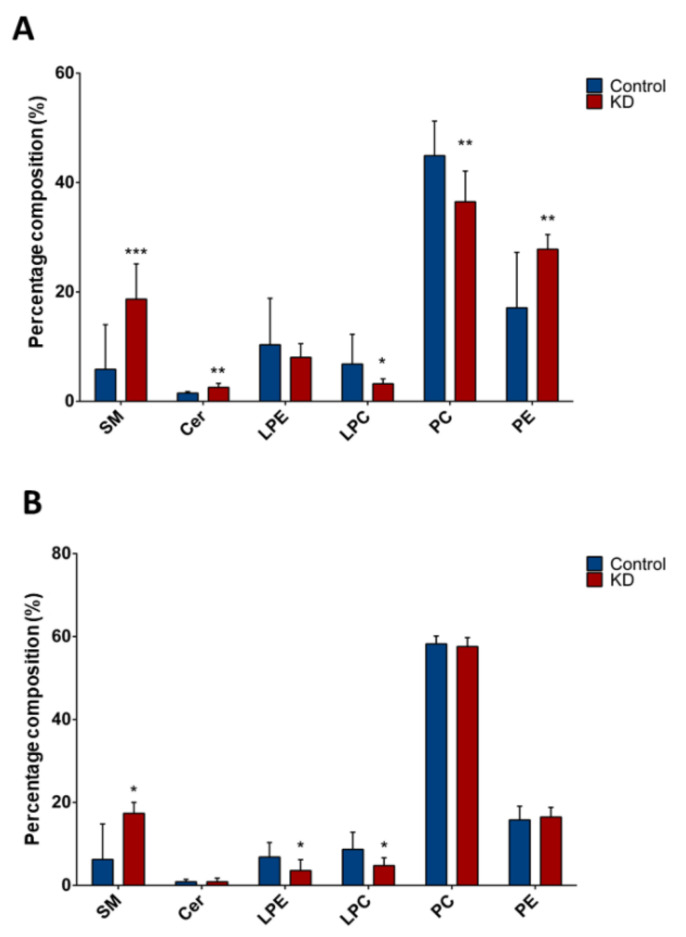
Composition of lipid classes in Huh-7/HepG2 cells depleted for macroH2A1. (**A**) Percentage composition of lipid classes in macroH2A1 KD Huh-7 cells (in red) and control cells (in blue). (**B**) Percentage composition of lipid classes in macroH2A1 KD HepG2 cells (in red) and control cells (in blue). Data are represented as median and interquartile range (IQR); * *p* < 0.05; ** *p* < 0.01; *** *p* < 0.001 based on Mann–Whitney test. SM, sphingomyelin; Cer, ceramides, LPE, lysophosphatidylethanolamines; LPC, lysophosphatidylcholines; PC, phosphatidylcholines; PE, phosphatidylethanolamines.

We sought to determine a potential lipid marker signature of liver CSCs by using high-throughput LC/MS-based lipidomics. Upon lipid extraction and LC/MS analyses, a mixture of individual species for Cer, SM, PC, PE, LPE, and LPC, varying the length of ceramide or fatty acid carbon chain, was obtained. We first analyzed the variation in lipid classes between Huh-7 or HepG2, depleted for macroH2A1 expression and their respective controls (Figure 2). For this purpose, we calculated the percentage composition (i.e., the percentage over total detected lipids) of each lipid class and compared it between different cell lines and conditions. Interestingly, Huh-7 and HepG2 presented similar lipid changes upon macroH2A1 depletion. Indeed, total SM levels displayed 2.8-fold increase in macroH2A1 KD Huh-7 cells (*p* < 0.02) and 3.2-fold increase in macroH2A1 KD HepG2 cells (*p* < 0.001) compared to control cells (Figure 2A,B). By contrast, we observed 0.5-fold decrease of total LPC in both macroH2A1 KD Huh-7 and macroH2A1 KD HepG2 cells compared to controls (*p*-values < 0.05) (Figure 2A,B). With respect to macroH2A1 KD Huh-7 cells, we detected a 1.7-fold increase of total ceramides levels (*p* = 0.006) and a 0.8-fold decrease of total PC levels (*p* < 0.001) (Figure 2A). We also found a 0.5-fold decrease of total LPE only in macroH2A1 KD HepG2 (*p* < 0.03) (Figure 2B).

**Figure 3:**
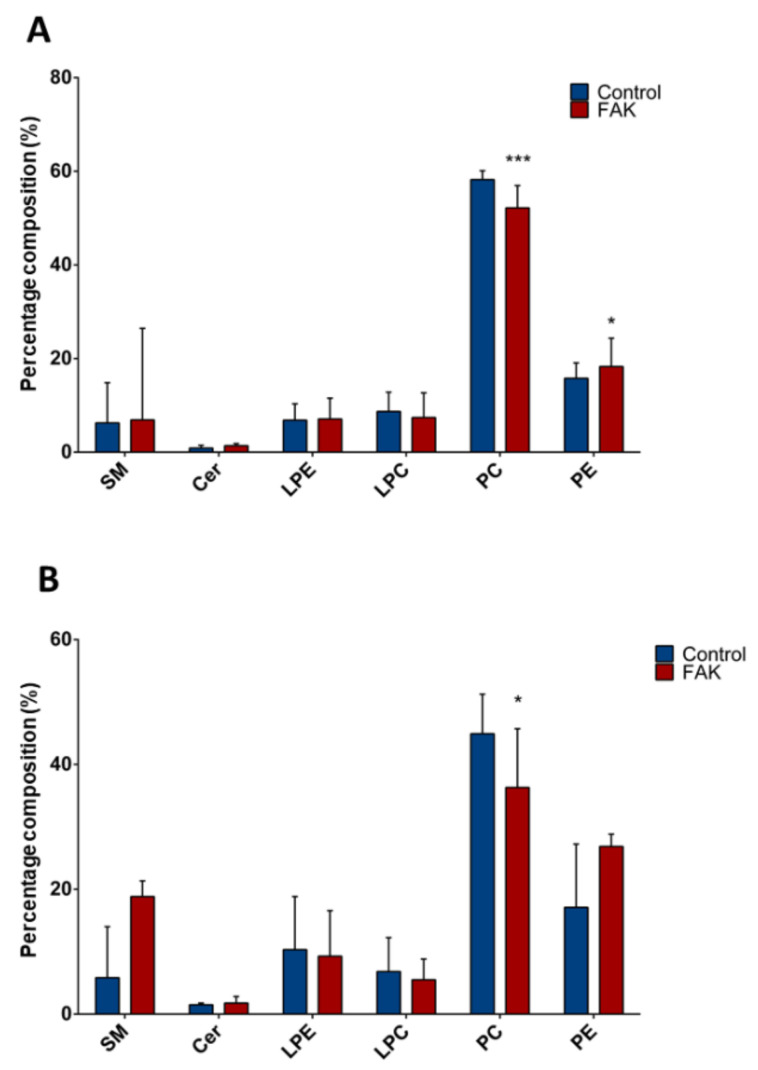
Composition of lipid classes in Huh-7/HepG2 cells depleted for FAK. (**A**) Percentage composition of lipid classes in KD Huh-7 cells (in red) and control cells (in blue). (**B**) Percentage composition of lipid classes in KD HepG2 cells (in red) and control cells (in blue). Data are represented as median and interquartile range (IQR); * *p* < 0.05; *** *p* < 0.001. SM, sphingomyelin; Cer, ceramides, LPE, lysophosphatidylethanolamines; LPC, lysophosphatidylcholines; PC, phosphatidylcholines; PE, phosphatidylethanolamines.

In the same way, we compared composition of lipid classes between FAK KD Huh-7 or HepG2 and their respective controls (Figure 3). In both Huh-7 and HepG2 cell lines depleted for FAK, we found ~0.8-fold decrease of total PC (*p* < 0.001 and *p* < 0.03, respectively; Figure 3A,B). In FAK KD Huh-7 cells, we also observed a 1.3-fold increase of total PE compared to control cells (*p* < 0.03; Figure 3A).

### 2.3. Changes in Specific Lipid Species in Huh-7 and HepG2 Cells Depleted for macroH2A1 or FAK

We next compared absolute levels of specific lipid species between Huh-7 or HepG2, depleted for macroH2A1 or FAK and their respective control cells. 

**Figure 4:**
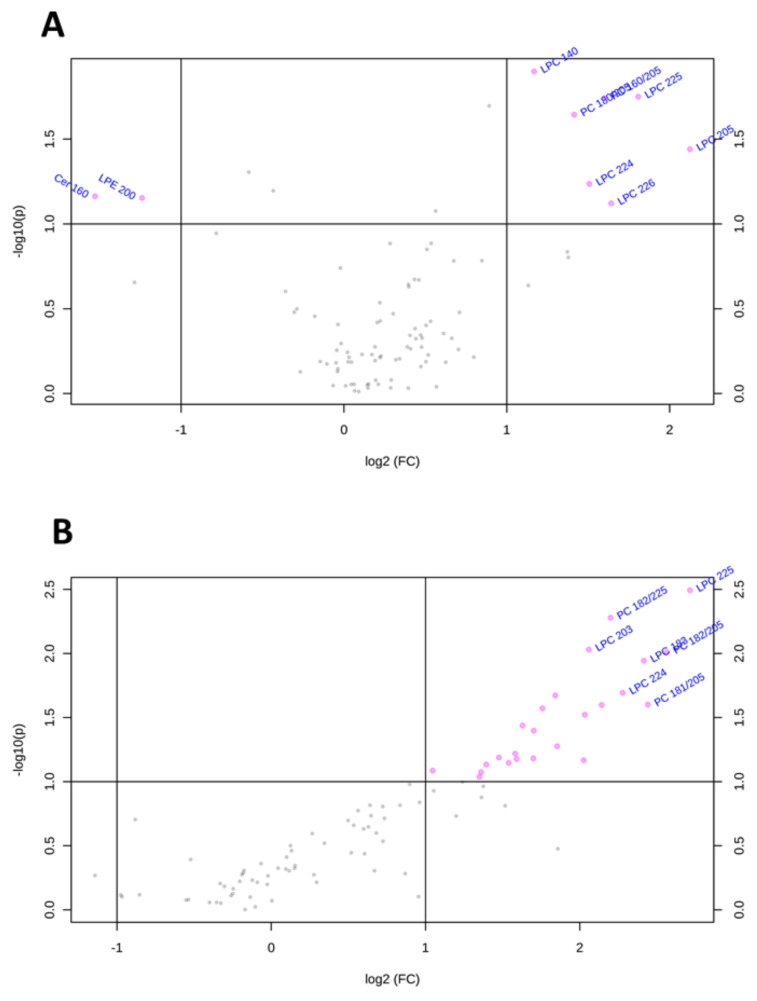
Changes of lipid species in Huh-7/HepG2 cells depleted for macroH2A1. (**A**) Important features selected by volcano plot with fold change threshold 2 and t-tests threshold 0.1 in KD Huh-7 cells. (**B**) Important features selected by volcano plot with fold change threshold 2 and t-tests threshold 0.1 in KD HepG2 cells. Note both fold changes and *p*-values are log-transformed.

To visualize the most important lipid features that characterize Huh-7 and HepG2 cells depleted for macroH2A1, we present the volcano plots shown in Figure 4, which summarize changes in specific lipid species on the basis of a fold change threshold of 2 and t-tests threshold of 0.1. Table 1 summarizes fold changes and *p*-values for each lipid species selected by the volcano plot. Specifically, Cer 16:0 and LPE20:0 were downregulated while LPC14:0, LPC22:4, LPC22:6, LPC22:5, LPC20:5, and PC 18:0/20:5 were upregulated in macroH2A1 KD Huh-7 cells (Figure 4A). In macroH2A1 KD HepG2, instead, we were able to identify a total of 32 upregulated lipids, out of which 8 exhibited a 5-fold increase or higher (i.e., LPC22:5, LPC20:3, LPC22:4, LPC18:3, PC18:2/22:5, PC 18:2/20:5, and PC18:1/20:5; Figure 4B). Thus, the upregulation of LPC22:4 and LPC22:5 is a common lipid feature that characterizes Huh-7 and HepG2 cells depleted for macroH2A1.

**Figure 5:**
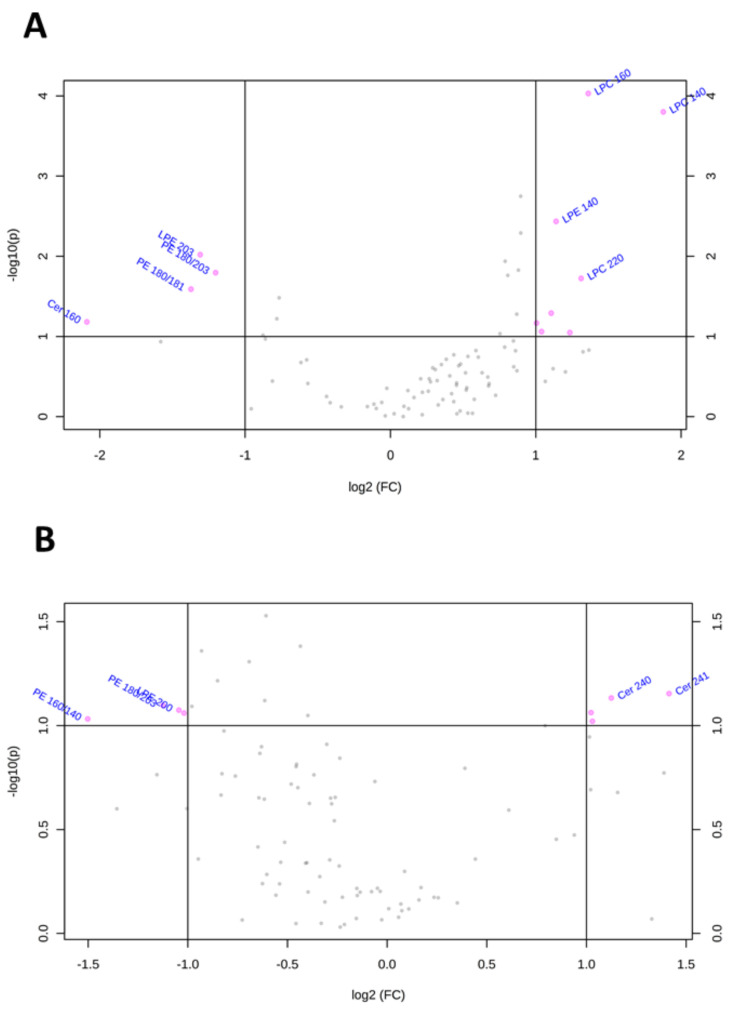
Changes of lipid species in Huh-7/HepG2 cells depleted for FAK. (**A**) Important features selected by volcano plot with fold change threshold 2 and t-tests threshold 0.1 in KD HuH-7 cells. (**B**) Important features selected by volcano plot with fold change threshold 2 and t-tests threshold 0.1 in KD HepG2 cells. Note both fold changes and *p*-values are log transformed.

Similarly, the volcano plots shown in Figure 5 and Table 2 summarize lipid species that were dysregulated in Huh-7 and HepG2 cells depleted for FAK. In FAK KD Huh-7 cells, the most important features included the downregulation of LPE20:3, PE18:0/20:3, PE18:0/18:1, and Cerd18:1/16:0 and the upregulation of LPE14:0, LPC22:0, LPC16:0, LPC14:0 (Figure 5A). Regarding FAK KD HepG2 cells, we observed the upregulation of Cer24:1, Cer24:0, SM18:0, and SM16:0 and the downregulation of PE18:0/20:3, PE16:0/20:3, PE16:0/14:0, and LPE20:0 (Figure 5B). Notably, none of the lipid features characterize both Huh-7 and HepG2 cells depleted for FAK.

The ratio of the main membrane phospholipid species, PC and PE, was also calculated (Table 3). The PC/PE ratio of FAK KD Huh-7 cells was significantly decreased by 1.6-fold (*p* = 0.01) compared to control cells, while the PC/PE ratio of FAK KD HepG2 was not altered compared to control cells. The PC/PE ratio of both cell lines in macroH2A1 KD Huh-7 and HepG2 cells was significantly decreased compared to control cells. The PC/LPC ratios of both cell lines in macroH2A1 KD Huh-7 and HepG2 cells were significantly increased compared to control cells, while in FAK KD Huh-7 and HepG2 cells, there was a decrease in PC/LPC ratio. Lipid desaturation of fatty acids is an essential feature of aggressive cancer stem-like cells [10,11,12,17,18,23,24,25]. We assessed total unsaturated fatty acid (UFA) levels in macroH2A1 KD or FAK KD Huh-7 and HepG2 cell lines, using an ELISA assay: UFA levels were significantly increased (*p* = 0.01) and not significantly increased (*p* = 0.09) in macroH2A1 KD Huh-7 and HepG2 cells, respectively (Table 3). Conversely, there was no difference in UFA levels in FAK KD cells (Table 3).

### 2.4. Lipid Marker Signature Discriminates Huh-7 Cells Depleted for macroH2A1 or FAK

**Figure 6:**
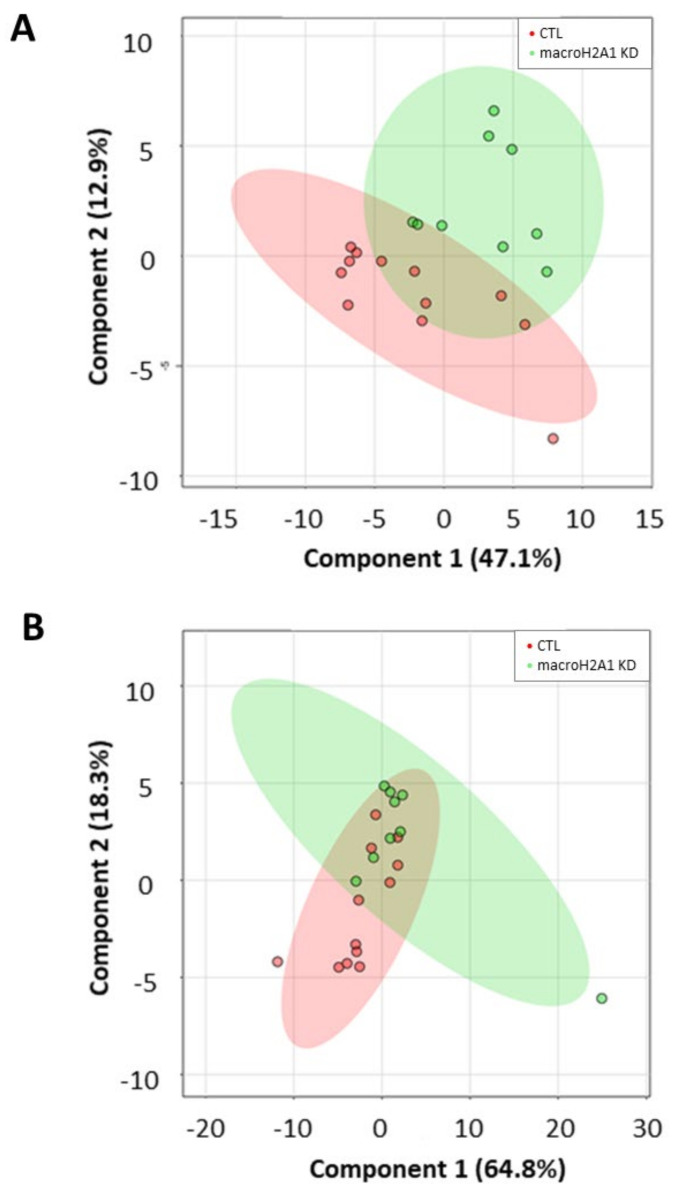
Discriminant ability of lipid signature in Huh-7/HepG2 cells depleted for macroH2A1. (**A**) Partial least squares discriminant analysis (PLS-DA) score plot showing separation between KD Huh-7 cells (green) and control cells (red). (**B**) PLS-DA score plot showing separation between KD HepG2 cells (green) and control cells (red). Number in parentheses is the percentage of explained variation.

**Figure 7:**
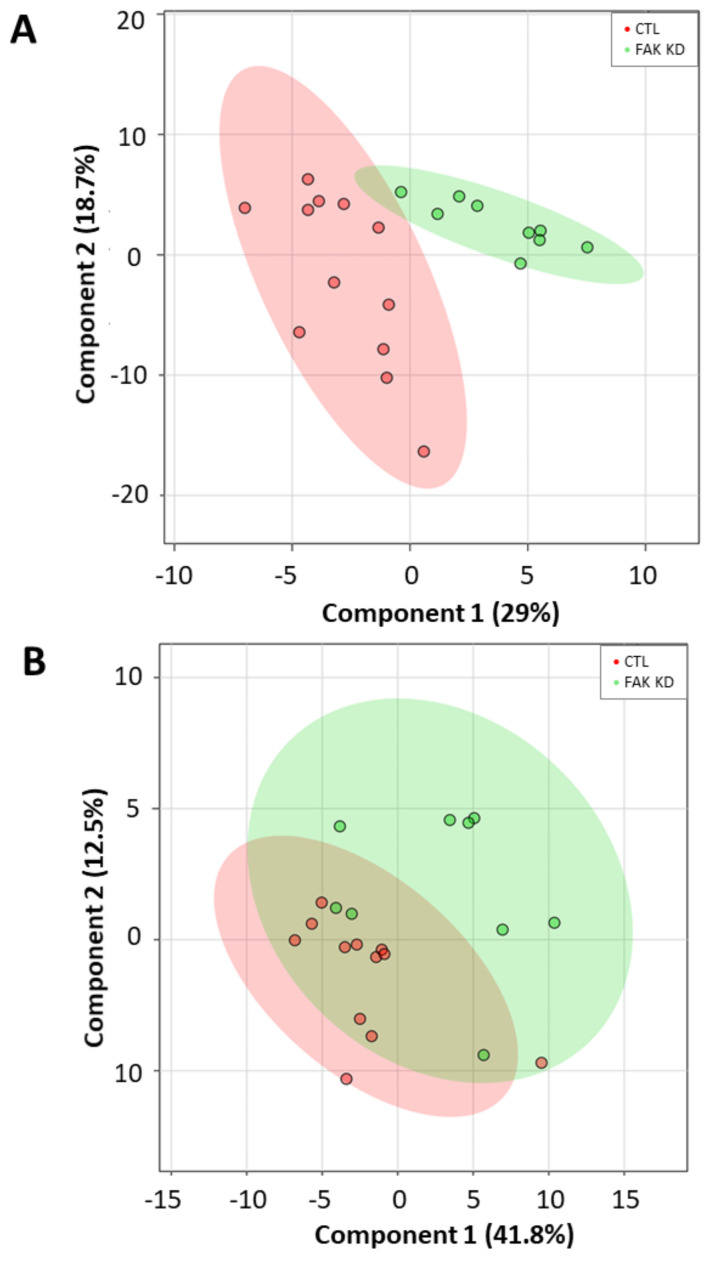
Discriminant ability of lipid signature in Huh-7/HepG2 cells depleted for FAK. (**A**) Partial least squares discriminant analysis (PLS-DA) score plot showing separation between KD Huh-7 cells (green) and control cells (red). (**B**) PLS-DA score plot showing separation between KD HepG2 cells (green) and control cells (red). Number in parentheses is the percentage of explained variation.

To evaluate whether a lipid marker signature could discriminate cells depleted for macroH2A1 or FAK from their respective controls, we next applied a principal component analysis (PCA) on absolute lipid values, a multivariate unsupervised method for data reduction and pattern discovery. However, no clear separation was evident either in cells depleted for macroH2A1 or in those depleted for FAK (Supplemental Figures S2–S5). To maximize the separation between different cell conditions, we finally applied a partial least squares discriminant analysis (PLS-DA), a multivariate supervised classification method useful to predict the class membership. With this purpose, we included in the PLS-DA all the absolute normalized log-transformed values of lipid species. Accordingly, the PLS-DA score plots reported in Figure 6 indicate a partial discrimination between macroH2A1 KD Huh-7 cells and control cells (Figure 6A). In contrast, no clear separation between macroH2A1 KD and control cells is evident for the HepG2 cell line (Figure 6B). With respect to cells depleted for FAK, the PLS-DA score plot in Figure 7A indicates a good discrimination in Huh-7 cells. In contrast, no clear separation between FAK KD and control cells is evident for the HepG2 cell line (Figure 7B). Performance measures obtained from the cross-validation are reported in Supplemental Figures S6–S9.

With respect to cells depleted for FAK, the PLS-DA score plot in Figure 7A indicates a good discrimination in Huh-7 cells. In contrast, no clear separation between FAK KD and control cells is evident for the HepG2 cell line (Figure 7B).

## 3. Discussion

Changes in lipid metabolism and cell/tissue composition play a crucial role in the development of HCC [26]. Previous studies have reported an increase in genes related to phospholipid metabolism in hepatocellular carcinoma [27]. Furthermore, alterations in lipid metabolism, such as lipid desaturation, were found to be intensively involved in CSCs generation and stemness maintenance [14,25]. Previous studies demonstrated that the loss of histone macroH2A1 in HCC is associated with poorly differentiated, more CSC-like, and more aggressive HCC, and it also results in abnormal triglyceride accumulation [17,18,19], whereas inhibition of FAK in HCC cells reduces tumorigenicity [21] and CSC-like phenotype [22].

We thus designed the current “oncolipidomic” study to analyze the difference in sphingolipid and phospholipid composition in in vitro HCC cells prompted to acquire CSC traits (upon macroH2A1 depletion) or with a suppressed stemness phenotype (upon FAK depletion). Our results showed that depletion of these genes caused abnormalities in the sphingolipid and phospholipid metabolism/composition by elevating or attenuating some individual lipid classes and/or species. The depletion of FAK had an impact on the main structural phospholipids decreasing total levels of PC and increasing total levels of PE (*p* < 0.05) but SM, Cer, LPE, and LPC were not significantly different (*p* > 0.05). The depletion of macroH2A1 had an impact on SM, Cer, LPC, LPE, but PE was not affected (*p* > 0.05), while total PC decreased significantly in macroH2A1 KD HepG2 cells (*p* < 0.05). Choline-containing lipids are generally involved in cell reprogramming [27]. In the liver, PC is synthesized from choline via the CDP-choline pathway or by methylation of PE via phosphatidylethanolamine N-methyltransferase (PEMT) [28]. PC can also be synthesized by acylation of LPC and the PC/PE ratio is a well-established indicator of membrane integrity [28], and abnormal alterations of this ratio have profound impact on systemic energy metabolism [29]. Interestingly, significantly decreased PC/PE ratios were identified in HCC cells upon depletion of macroH2A1. Low PC/PE ratio is associated with disrupted membrane integrity in the liver [28]; moreover, increased PE levels may increase autophagic flux, which we previously reported in both HepG2 and Huh-7 cells depleted for macroH2A1 [18]. In HCC, overexpression of LPC acyltransferase 1 catalyzes the conversion of LPC into PC in the Lands cycle of PC biosynthesis, promoting cell proliferation [26]. In the present study, the loss of macroH2A1, but not FAK, in HCC cells decreased total levels of both PC and LPC lipid classes, which might be consistent with the observed decrease in cell proliferation, resembling the quiescent state typically observed in CSCs [17,18,19].

The role of fatty acids, such as palmitic acid (PA, C16:0) fatty acid (palmitic acyl) in HCC progression and stemness acquisition is controversial. In HCC cells, C16:0 supplementation restored PA-containing lipids and reduced cell migration and invasiveness due to reduced activation of glucose transport carrier proteins by limiting glucose utilization [9]. Some other studies indicate that PA is lipotoxic and modulates the progression of nonalcoholic steatohepatitis [30]. In this respect, recent studies identified circulating and liver tissue PC16:0 and 18:0 lipid species from patients with cirrhosis and HCC to predict worse outcome and mortality [31,32]. We found a significant increase in some saturated PC lipid species (e.g., 16:0, 18:0) as well in macroH2A1 KD HCC cells; however, this effect was absent in FAK KD HCC cells (Table 1 and Table 2). In fact, our data show that the depletion of FAK in Huh-7 cells decreased levels of several PA derivatives, consistent with their opposite cancer aggressiveness and stemness features. Our findings show also that inhibition of FAK in HCC cells might confer protective anticancer effects by decreasing the levels of long-chain Cer (24:0), which is instead upregulated in human HCC and associated with patient survival [31,32]. While total LPC and LPE levels did not vary upon FAK KD in HCC cells (Figure 2), some saturated species of LPC (14:0; 16:0) and LPE (14:0) were massively and significantly upregulated in Huh-7 cells (Table 2): of note, these species have been reported to be significantly decreased in the sera of HCC patients in multiple publications [33]. This could be further consistent with the anticancer and antistemness effects of FAK depletion.

Our PLS-DA uncovered that the observed differences in lipidomic species upon macroH2A1 or FAK KD are better discriminated in Huh-7 compared to HepG2 cells (Figure 6 and Figure 7). HepG2 cells express a wild-type tumor suppressor p53, while Huh-7 cells express a mutant dominant negative p53 (Y220C) [34]. Mutations in the p53 gene are frequent in human HCC and are associated with a shorter survival rate compared to the one of wild-type p53 carriers [35]; they are also major components in the establishment of CSC entity [36]. Ricchi et al. showed that the absolute ability to accumulate triglycerides is much greater in Huh-7 cells compared to HepG2 cells [37], and the two cell lines display significant differences in glycolytic pathways and in their secretomes [17,19]. Altogether, these findings suggest that the Huh-7 cell line might be more prone to model CSCs in vitro.

Another factor that may contribute to abnormalities is the incorporation of fatty acids into phospholipids, which is mediated by lysophospholipid acyltransferases in the Kennedy pathway and/or Land’s cycle and requires acyl-CoA [38]. Recently, we have shown that acetyl-CoA in macroH2A1-depleted CSC-like cells is overexpressed, which affects acyl-CoA and fatty acid synthesis [17]. 

Finally, we found that macroH2A1 KD HCC cells display higher levels of unsaturated fatty acid species compared to control cells. Interestingly, Li et al. (2017) have recently shown that unsaturated fatty acids maintain breast cancer cell stemness via NF-κB activation [23]. All these findings are consistent with our previous studies on unsaturated fatty acids, NF-κB activation, and HCC aggressiveness [10,11,12,17,18], suggesting that therapeutic targeting of fatty acid unsaturation might be effective for selective elimination of liver CSCs [24,25].

## 4. Materials and Methods

### 4.1. Cell Cultures

The HepG2 and Huh-7 parental cell lines (ATCC) were cultured in DMEM (1X) supplemented with 10% fetal bovine serum (FBS) and 1% penicillin/streptomycin. Stable knockdown (KD) of macroH2A1 or PTK2 (FAK) was achieved by lentiviral transduction, as previously described [17,18,19,21]. For lipidomic experiments, experiments were conducted utilizing three cellular replicates, and the experiments were performed 3–4 times at different cellular passage numbers.

### 4.2. Extraction of Lipids from HCC Cell Pellets

Total lipids were extracted using the modified Folch method [39]. Briefly, cell pellets containing Equisplash™ Lipidomix^®^ as deuterated lipid internal standard (330731-1EA, Avanti Polar Lipids, Alabaster, AL, USA) were mixed with chloroform/methanol (2:1, *v/v*) and 0.025% of CaCl_2_ and shaken with a thermoshaker (TS-100, Biosan, Riga, Latvia) at 1400 rpm for 20 min. After shaking, cell pellets were sonicated for 2 min (Kraintek 18). After centrifugation (16000× *g*, 15 min, 4 °C; Eppendorf 5427R), the lower phase was extracted and the top layer was processed again following the same Folch extraction. Layers were combined and were evaporated using a speed vacuum. Lipid extracts were reconstituted with ACN:H_2_O (3:1, *v/v*).

### 4.3. Liquid Chromatography/Mass Spectrometry (LC/MS) Analysis

Lipid extraction was analyzed using a UPLC system composed of a Thermo Scientific Dionez UltiMate™ 3000 RSLCnano system connected to a ABSciex QTRAP 6500 system. A Waters Acquity UPLC BEH HILIC, 1.7 um, 2.1 × 100 mm (Waters, Milford, MA, USA) column with a guard column, Acquity UPLC BEH HILIC 1.7 μm VanGuard Pre-Column 2.1 × 5 mm (Waters, Milford, MA, USA), was used for lipid separation. The solvent gradient was prepared as follows: a linear gradient from 0 to 10 min to 80% mobile phase A (water/acetonitrile [5:95, *v/v*] with 10 mM ammonium acetate) was held and over 11 min to 98% mobile phase B (water/acetonitrile [50:50, *v/v*] with 10 mM ammonium acetate; pH = 8.2) to return to the initial conditions after 2 min. The flow rate through the column was 400 μL/min. Mass spectrometry analyses were performed using electrospray ionization and multiple reaction monitoring (MRM) scans (Table S1). A library of theoretical precursor ions was generated for Cer, SM, PC, PE, LPE, and LPC varying the length of ceramide or fatty acid carbon chain. The characterization of phospholipid and sphingolipid classes and individual species was achieved in positive mode (Cer and SM) with the formation of [M + H]^+^ and in negative mode (PE, LPE, LPC) with the formation of [M−H]^−^ and [M + OAc]^−^ for PC. A library of the probable FA carbon chains present in these phospholipid species (PE, PC, LPC, LPE) was developed and used as product ions. The collision energy varied from 40 to 50 eV. MS additional parameters were as follows: curtain gas (CUR) 35; temperature (TEM) 500 °C; Ion Source Gas 1 (GS1) 40; Ion Source Gas 2 (GS2) 50; Ion spray voltage 5200/−4500.

### 4.4. Transcriptomic Analyses

Total RNA was extracted from three biological replicates of control and macroH2A1 KD HepG2 or Huh-7 cells with TRIzol Reagent (Invitrogen, Vienna, Austria). Indexed libraries were prepared from 2 mg each purified RNA using a TruSeq Total Stranded RNA Sample Prep Kit (Illumina, Cambridge, UK) according to the manufacturer’s instructions. Libraries were quantified on an Agilent 2100 Bioanalyzer (Agilent Technologies, Vienna, Austria) and pooled so that each index-tagged sample was present in equimolar amounts; the final concentration of the pooled samples was 2 nmol/L. Pooled samples were then subjected to cluster generation and sequenced on an Illumina HiSeq 2500 System (Illumina) in a 2 × 100 paired-end format at a final concentration of 8 pmol/L.

### 4.5. Open Array

A predesigned TaqMan OpenArray Human Cancer Panel (Life Technologies, Thermo Fisher Scientific Corporation, Foster City, CA, USA) was used to assess the effect of FAK inhibition in HepG2 and Huh7cells on a signature panel of 609 well-defined genes validated for the characterization of undifferentiated stem cells or their differentiated derivatives, plus 22 endogenous control genes. cDNAs were loaded onto the Open Array platform and run as recommended by the manufacturer on the QuantStudio 12K Flex Real-Time PCR system (Life Technologies, Thermo Fisher Scientific corporation). Relative gene expression values were calculated as relative quantity (RQ) by using Open Source expression suite provided by Life Technologies. RQ minimum and maximum values (error bars) were calculated with a confidence level of 95%, using Benjamini–Hochberg false discovery rate to adjust *p* values. Maximum allowed Ct included in calculations was 35. Multivariate Student’s t-test or one-way ANOVA was applied and values of *p* < 0.05 were considered statistically significant.

### 4.6. Lipid Quantification Assays

Total unsaturated fatty acids were assessed using the Lipid Assay Kit from Abcam (ab242305, Prague, Czech Republic), according to manufacturer’s instructions.

### 4.7. Bioinformatic Analyses

RNA-Seq short-reads were aligned against the hg19 genome assembly using STAR (ver. 2.6) with standard parameters, considering the genome features extracted from the UCSC RefSeq gtf file. Piled-up reads were counted using the htseq-count tool [40]. Counts were normalized (counts per million, CPM) and compared between the two contrasts (KD) and (control) using the edgeR R package (ver. 3.7). Genes were considered differentially expressed between groups if their expression values significantly differed by > 2-fold and *p* ≤ 0.05. Data are available in Gene Expression Omnibus (GEO) with the accession numbers GSE117459 (for HepG2 cells) and GSE131680 (for Huh-7 cells) [17,18,19]. An unbiased list of genes functionally related to the cancer stemness and reprogramming biological functions was retrieved using the Ingenuity Pathway Analysis (IPA, spring 2018 release, QIAGEN Inc., Hilden, Germany, https://www.qiagenbioinformatics.com/products/ingenuity-pathway-analysis) software package. All metabolites exhibiting any significant (*p* < 0.05) effect size were considered differentially expressed. All analyses were performed in R ver. 3.4.2 (R Development Core Team 2017).

### 4.8. Statistical Analyses

To assess the variation of lipid classes between treated cells and controls, data are expressed as percentage composition, which correspond to the percentage of each lipid class over all the detected lipids. Due to the skewness, percentage composition of lipid classes is expressed as median and interquartile range and compared using the Mann–Whitney U test. Changes in lipid classes are also reported in terms of fold change in KD cells and using control cells as reference group. With respect to lipid species, instead, data are reported as absolute values that were normalized to the internal standard, log-transformed, and standardized using the z-scores. To select the most important lipid features that characterize KD cells, volcano plots were generated according to fold-change threshold of 2 and t-tests threshold of 0.1. To evaluate whether lipid signature could discriminate KD cells from the respective controls, we also applied principal component analysis (PCA) and partial least squares discriminant analysis (PLS-DA). The first one is an unsupervised method for data reduction and clustering, which projects the data into a new coordinate system along the principal component axes. The second one is a supervised method, which uses multivariate regression techniques to extract the information that can predict the class membership. To address the potential overfitting associated with PLS-DA, we carried out a leave-one-out cross-validation (LOOCV) determining three common performance measures: the sum of squares captured by the model (R2), the cross-validated Q2, and the prediction accuracy [41]. All the analyses were conducted using MetaboAnalyst 4.0, with a significance level of 0.05 unless indicated.

## 5. Conclusions

In conclusion, here we have identified specific lipidomic signatures that, under the control of key signaling/epigenetic pathways, hallmark malignant phenotype and stemness in HCC cells. Overall, in both HCC cell lines analyzed, macroH2A1 KD (which induces stemness) increased total SM levels and decreased total LPC levels, while FAK KD (which has antitumorigenic effects) decreased total PC levels. These findings were accompanied as well by cell-line-dependent and individual lipid species changes.

These data add another piece to the comprehension of the nexus between lipids species and stemness in liver cancer that may be applied for therapeutic purposes in future studies.

## Figures and Tables

**Table 1:** Important lipid features in Huh-7/HepG2 cells depleted for macroH2A1.

Lipid Species	Fold Change	*p*-Value
**Huh-7 Cells Depleted for macroH2A1**		
LPC (14:0)	2.3	0.013
LPC (22:5)	3.5	0.018
PC (16:0/20:5)	3.0	0.018
PC (18:0/20:5)	2.7	0.023
LPC (20:5)	4.4	0.036
LPC (22:4)	2.8	0.058
Cer (16:0)	0.4	0.069
LPE (20:0)	0.4	0.070
LPC (22:6)	3.2	0.076
**HepG2 Cells Depleted for macroH2A1**		
LPC (22:5)	6.6	0.003
PC (18:2/22:5)	4.6	0.005
LPC (20:3)	4.2	0.009
PC (18:2/20:5)	5.9	0.010
LPC (18:3)	5.3	0.011
LPC (22:4)	4.9	0.020
LPC (20:5)	3.6	0.021
PC (18:1/20:5)	5.4	0.025
PC (18:1/22:5)	4.4	0.025
PC (18:1/22:4)	3.4	0.027
PC (18:2/22:6)	4.1	0.030
LPC (20:1)	3.1	0.036
LPC (20:2)	3.3	0.040
LPC (18:2)	3.6	0.052
PC (18:0/20:5)	3.0	0.060
LPC (14:0)	2.8	0.065
LPC (18:1)	3.2	0.066
PC (16:0/20:5)	3.0	0.067
LPC (20:4)	4.1	0.068
LPC (16:1)	2.9	0.071
PE (18:1/22:4)	2.6	0.074
PC (16:0/22:4)	2.1	0.082
PC (16:1/18:2)	2.6	0.084
LPE (22:4)	2.6	0.092

Lipid species with fold change threshold 2 and t-tests threshold 0.1 were selected.

**Table 2:** Important lipid features in Huh-7/HepG2 cells depleted for FAK.

Lipid Species	Fold Change	*p*-Value
**Huh-7 Cells Depleted for FAK**		
LPC (16:0)	2.5	<0.001
LPC (14:0)	3.5	<0.001
LPE (14:0)	2.2	0.002
LPE (20:3)	0.4	0.006
PE (18:0/20:3)	0.5	0.015
LPC (22:0)	2.4	0.018
PE (18:0/18:1)	0.4	0.026
LPC (16:1)	2.1	0.063
Cer (16:0)	0.2	0.065
LPC (20:5)	2.0	0.070
PE (18:1/22:4)	2.1	0.082
LPC (20:1)	2.4	0.094
**HepG2 Cells Depleted for FAK**		
Cer (24:1)	2.7	0.070
Cer (24:0)	2.2	0.073
PE (18:0/20:3)	0.5	0.080
LPE (20:0)	0.5	0.084
SM (18:0)	2.0	0.087
PE (16:0/20:3)	0.5	0.087
PE (16:0/14:0)	0.4	0.093
SM (16:0)	2.0	0.095

Lipid species with fold change threshold 2 and t-tests threshold 0.1 were selected.

**Table 3:** Evaluation of PC/PE and PC/LPC ratios, UFA levels (mg/dL) in macroH2A1 KD and FAK KD cell lines.

	PC/PE	*p*-Value	PC/LPC	*p*-Value	UFA	*p*-Value
CTL Huh-7	4 ± 0.30	-	8 ± 1.43	-	158 ± 12	-
CTL HepG2	2 ± 0.30	-	11 ± 2.71	-	174 ± 19	-
macroH2A1 KD Huh-7	3 ± 0.20	0.01	13 ± 2.03	0.03	231 ± 21	0.01
macroH2A1 KD HepG2	1 ± 0.11	0.01	15 ± 3.47	0.02	194 ± 10	0.09
FAK KD Huh-7	2.5 ± 0.23	0.001	6.5 ± 0.83	0.6	161 ± 36	-
FAK KD HepG2	2 ± 0.16	0.21	7 ± 0.74	0.06	169 ± 17	-

Data were expressed as means ± SEM.

## Supplementary Materials

The following are available online at https://www.mdpi.com/1422-0067/21/22/8452/s1. Figure S1. Changes in HepG2 and Huh-7 cells upon knock-down of macroH2A1 or FAK. Figure S2. Principal Component Analysis (PCA) plots showing no clear separation between Huh-7 cells depleted for macroH2A1 (KD; in green) and control cells (C; in red). Figure S3. Principal Component Analysis (PCA) plots showing no clear separation between HepG2 cells depleted for macroH2A1 (KD; in green) and control cells (C; in red). Figure S4. Principal Component Analysis (PCA) plots showing no clear separation between Huh-7 cells depleted for FAK (KD; in green) and control cells (C; in red). Figure S5. Principal Component Analysis (PCA) plots showing no clear separation between HepG2 cells depleted for FAK (KD; in green) and control cells (C; in red). Figure S6. PLS-DA cross validation result for Huh-7 cells depleted for macroH2A1. Figure S7. PLS-DA cross validation result for HepG2 cells depleted for macroH2A1. Figure S8. PLS-DA cross validation result for Huh-7 cells depleted for FAK. Figure S9. PLS-DA cross validation result for HepG2 cells depleted for FAK. Table S1. MRM-profiling method in positive and negative ion mode.
