# Supplementary material for: A Lipidomic Signature Complements Stemness Features Acquisition in Liver Cancer Cells
Source: Int J Mol Sci. 2020 Nov 10;21(22):8452. doi: 10.3390/ijms21228452 (PMC7709039; doi:10.3390/ijms21228452)
Supplement: Supplementary file 1 [file ijms-21-08452-s001.zip › ijms-982637-supplementary.docx]

Supplementary Material


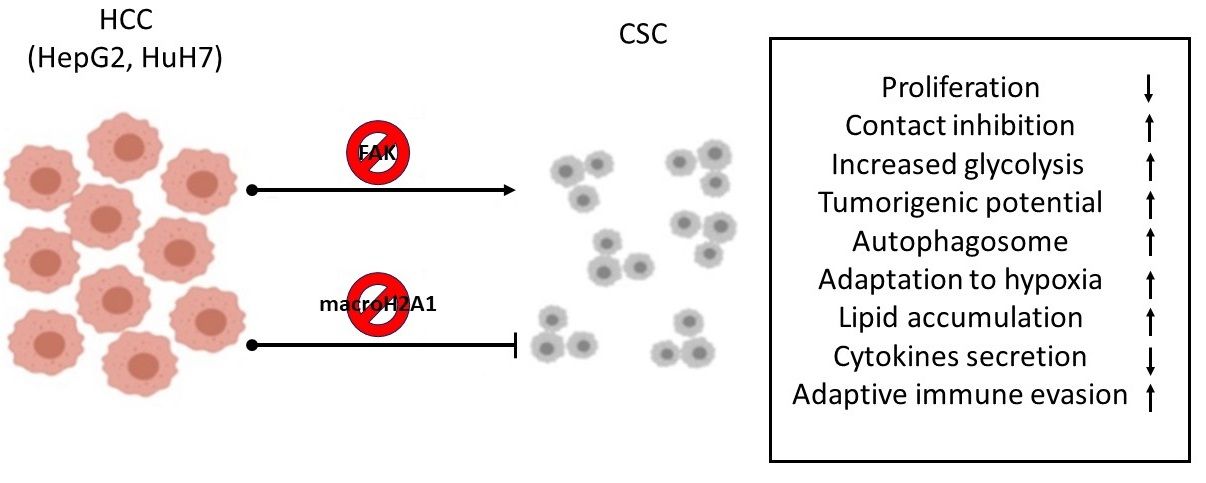


Supplemental Figure S1. Changes in HepG2 and Huh-7 cells upon knock-down of macroH2A1 or FAK


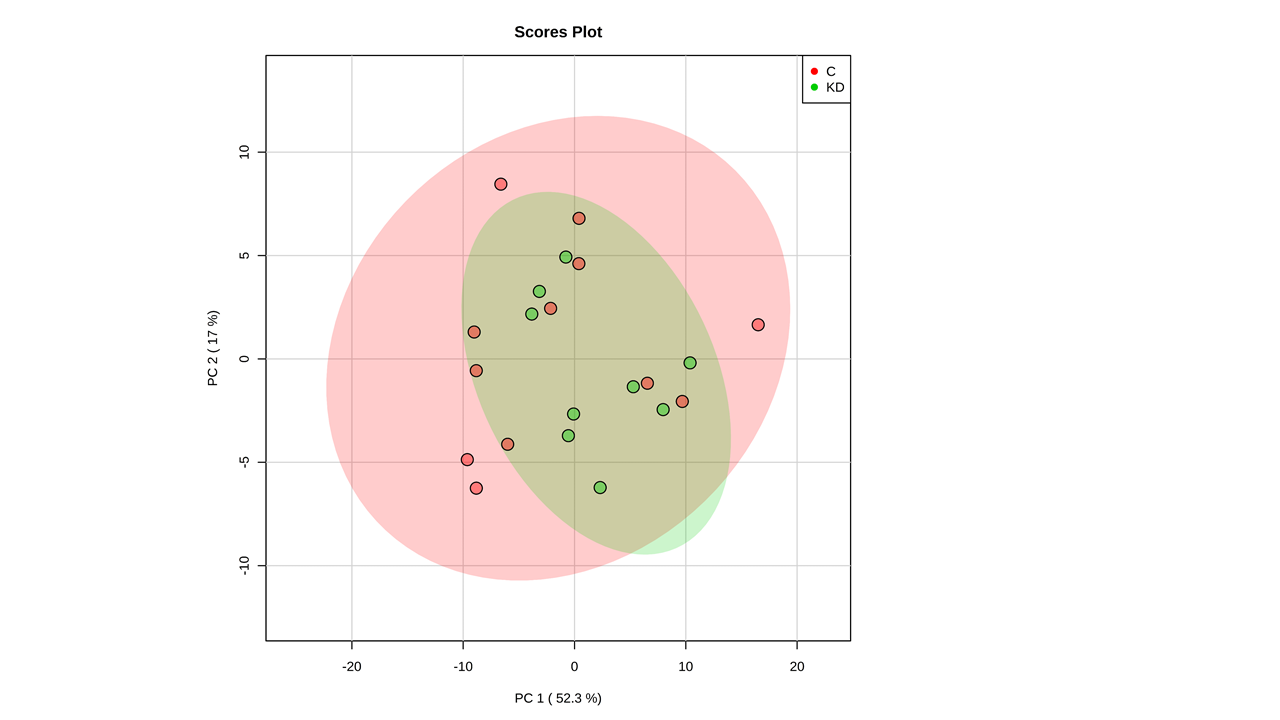


**Supplemental** **Figure S2.** Principal Component Analysis (PCA) plots showing no clear separation between Huh-7 cells depleted for macroH2A1 (KD; in green) and control cells (C; in red). Number in parentheses is the percentage of explained variation.


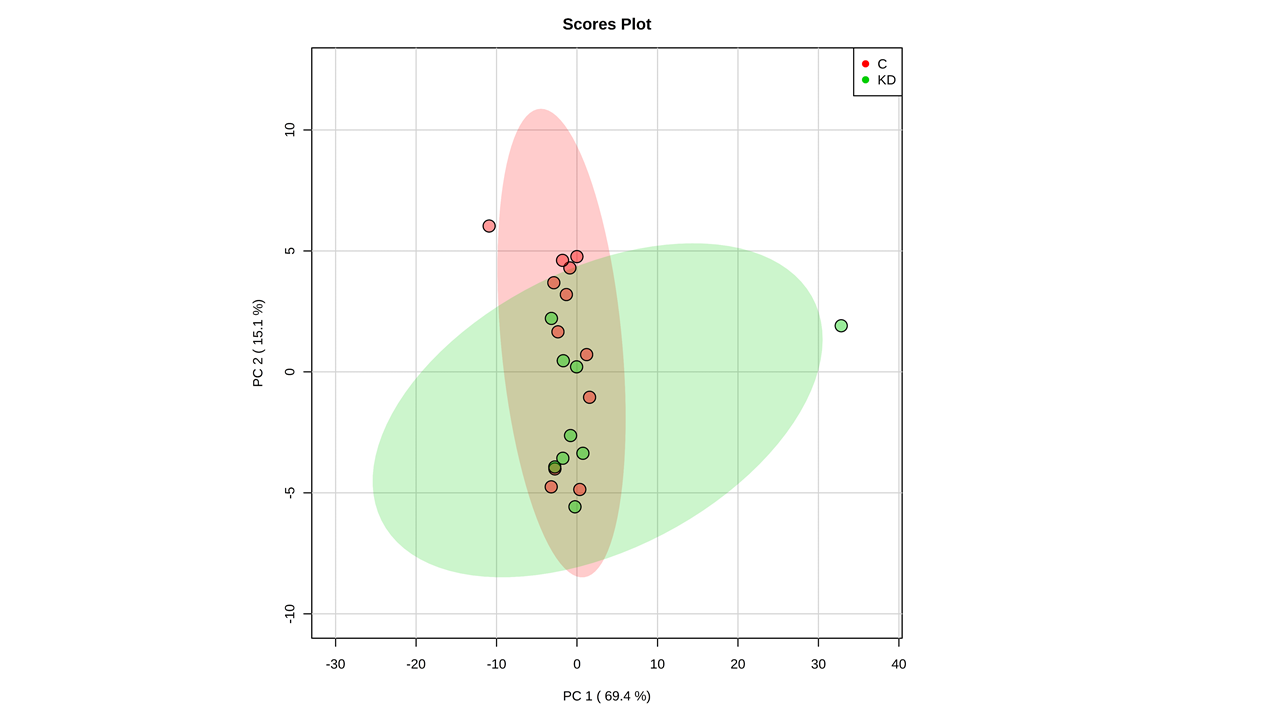


**Supplemental Figure** **S3.** Principal Component Analysis (PCA) plots showing no clear separation between HepG2 cells depleted for macroH2A1 (KD; in green) and control cells (C; in red). Number in parentheses is the percentage of explained variation.


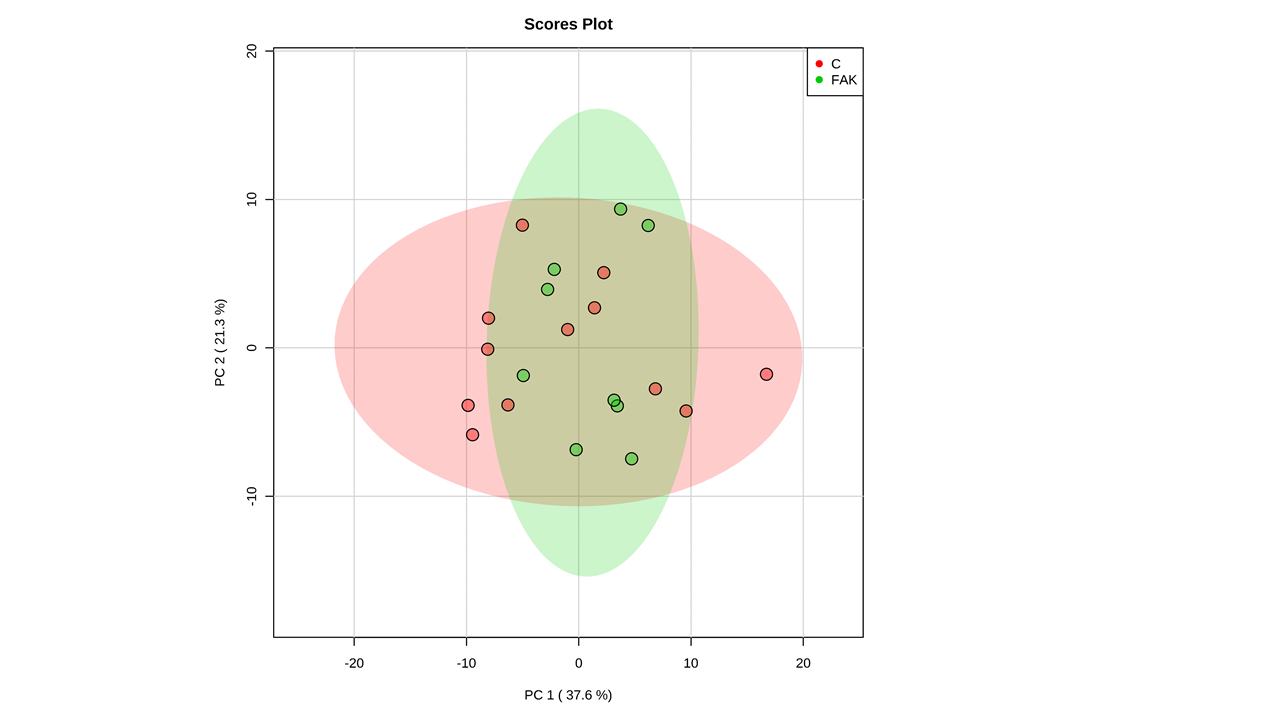


**Supplemental** **Figure S4.** Principal Component Analysis (PCA) plots showing no clear separation between Huh-7 cells depleted for FAK (KD; in green) and control cells (C; in red). Number in parentheses is the percentage of explained variation.


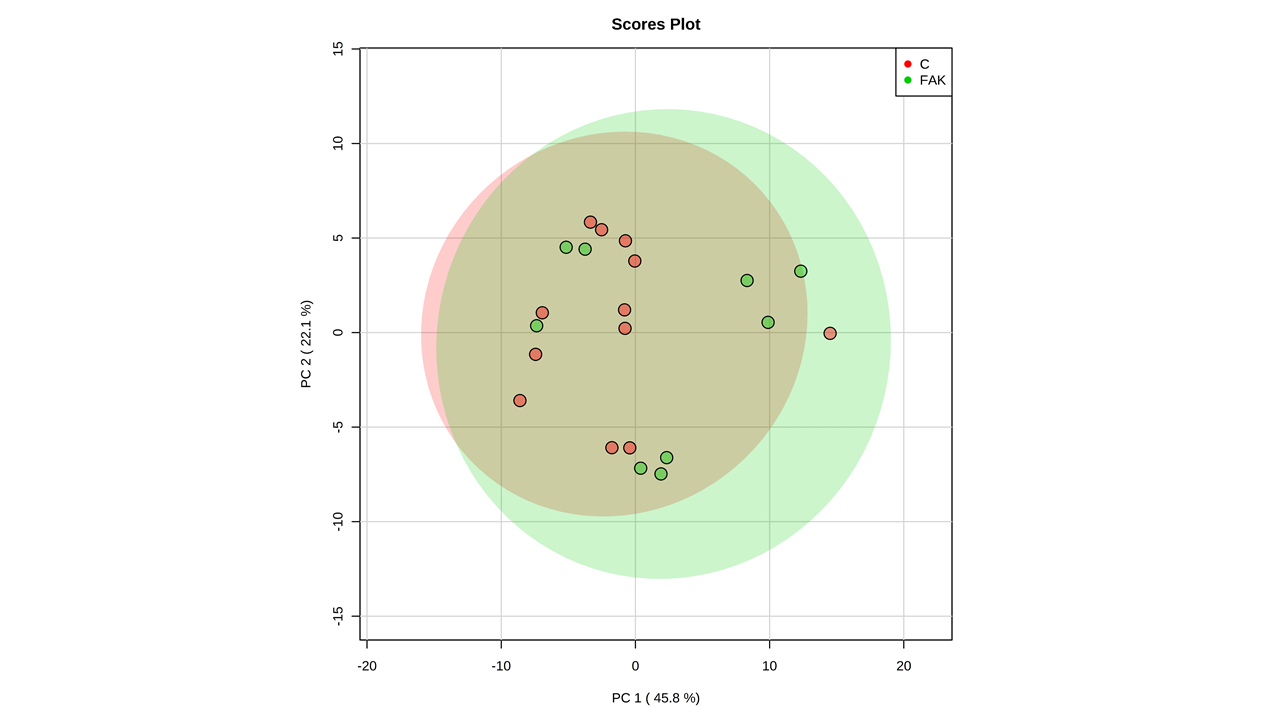


**Supplemental** **Figure S5.** Principal Component Analysis (PCA) plots showing no clear separation between HepG2 cells depleted for FAK (KD; in green) and control cells (C; in red). Number in parentheses is the percentage of explained variation.


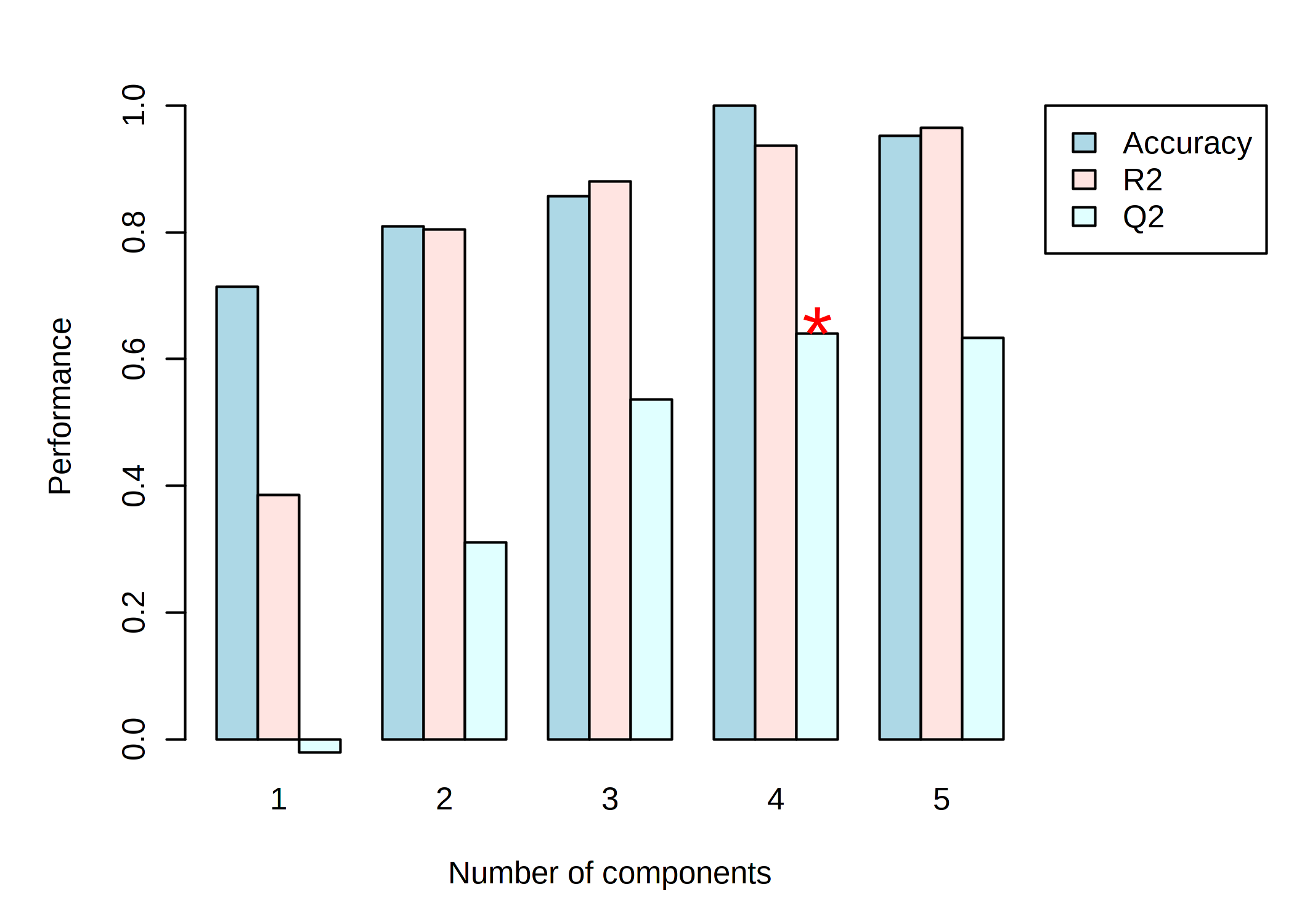


**Supplemental** **Figure S6.** PLS-DA cross validation result for Huh-7 cells depleted for macroH2A1. The selected performance measure (Q2) shows that the four-component model is best (indicated by a red star).


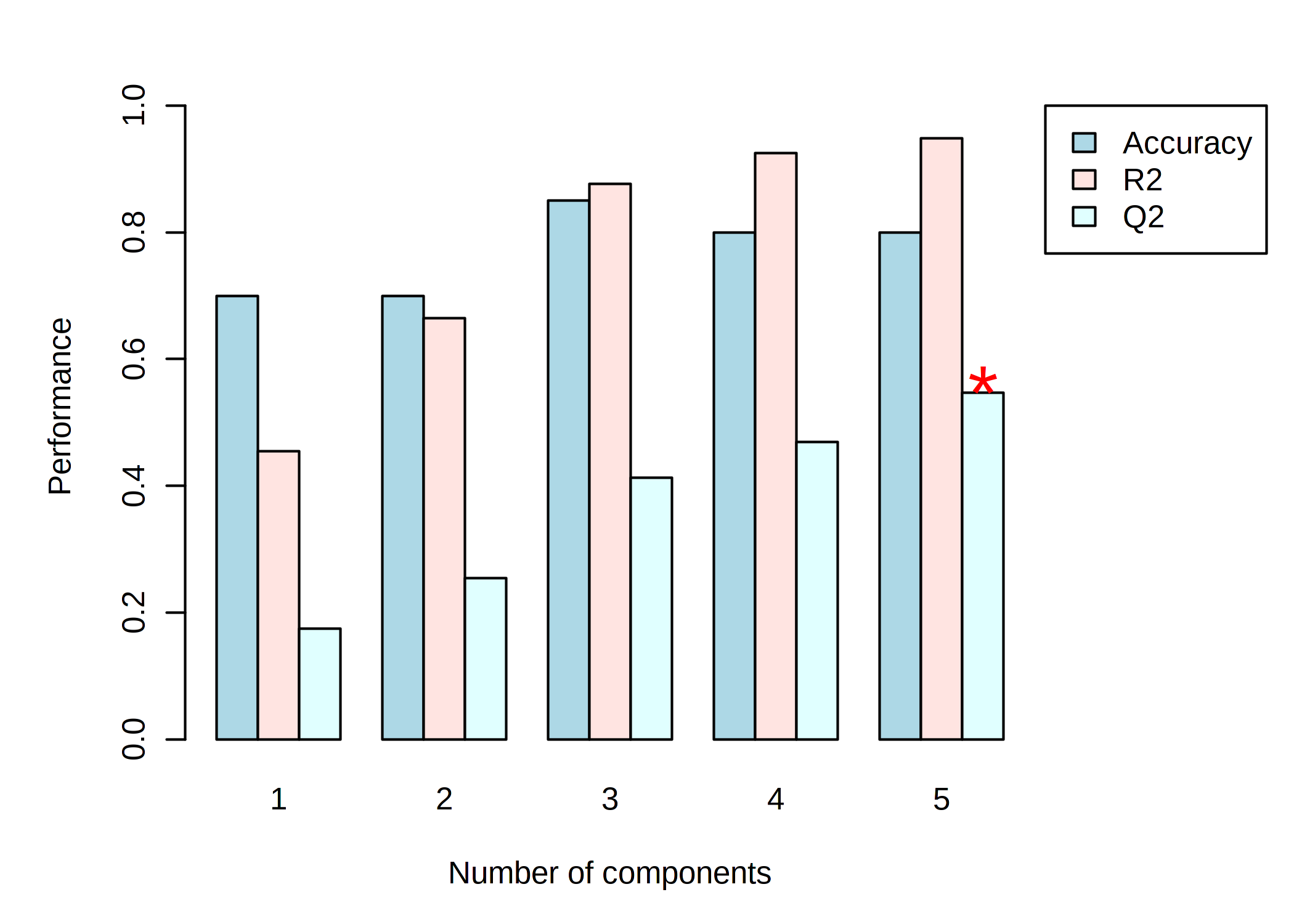


**Supplemental** **Figure S7.** PLS-DA cross validation result for HepG2 cells depleted for macroH2A1. The selected performance measure (Q2) shows that the five-component model is best (indicated by a red star).


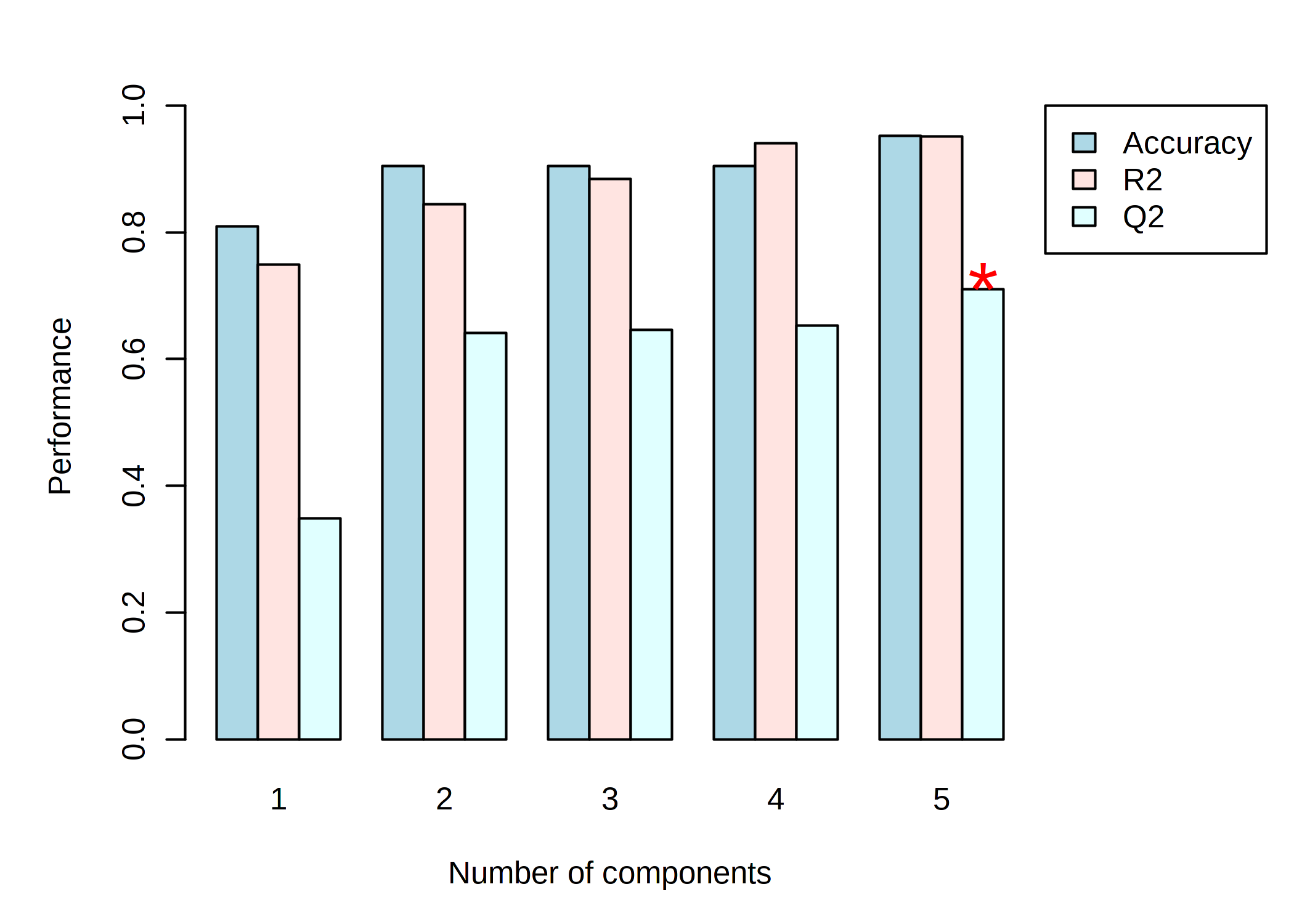


**Supplemental** **Figure S8.** PLS-DA cross validation result for Huh-7 cells depleted for FAK. The selected performance measure (Q2) shows that the five-component model is best (indicated by a red star).


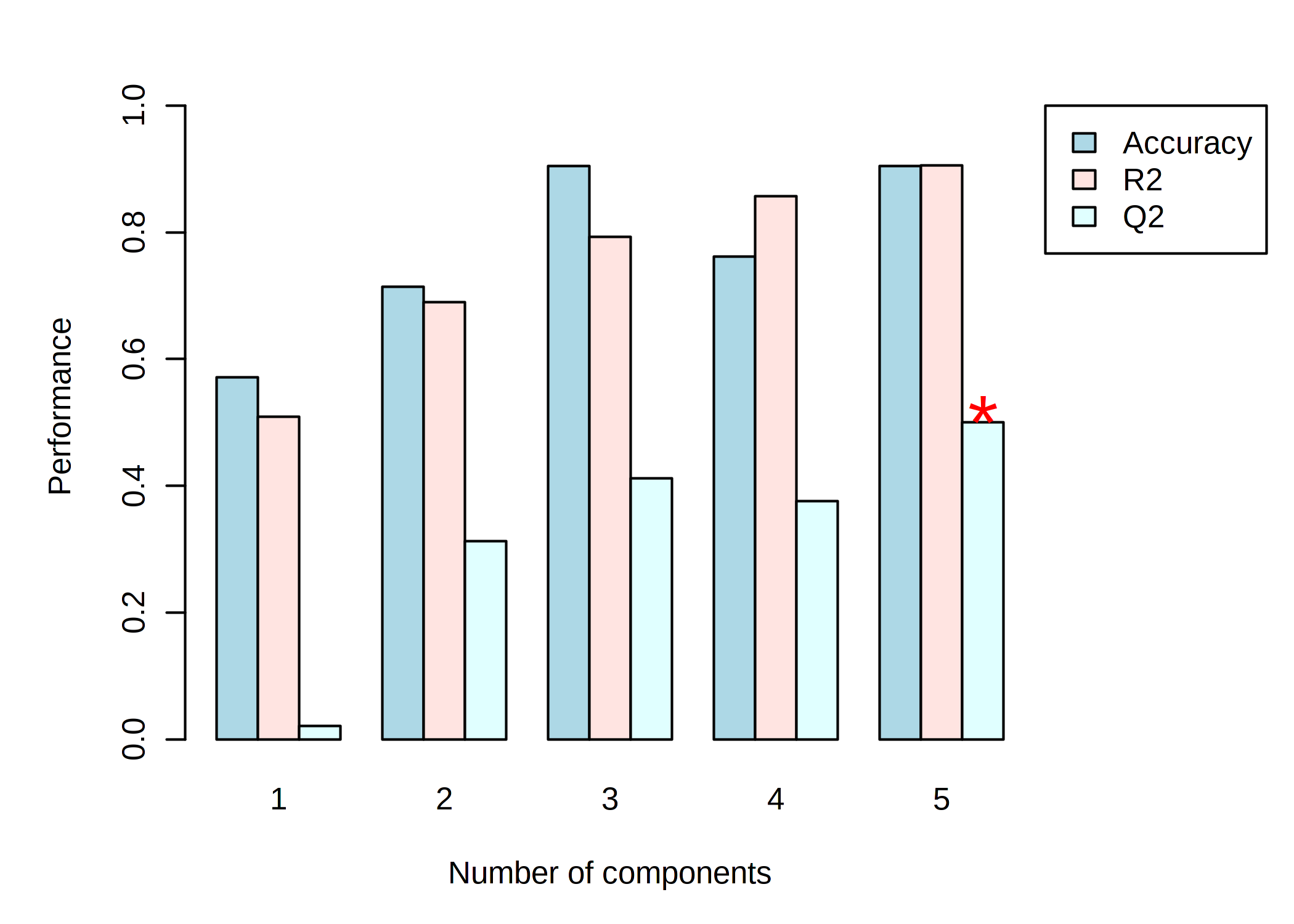


**Supplemental** **Figure S9.** PLS-DA cross validation result for HepG2 cells depleted for FAK. The selected performance measure (Q2) shows that the five-component model is best (indicated by a red star).

**Supplemental** **Table S1.** MRM-profiling method in positive and negative ion mode. List of transitions in the MRM profiling method in positive and negative ion mode of the lipid species detected in Huh-7 and HepG2 cells.

| Lipid Component | Detected ion type | Parent | Fragment | RT (min) | CE (eV) |
| --- | --- | --- | --- | --- | --- |
| Cer(d18:1/14:0) | [M+H]^+^ | 510.60 | 264.40 | 1.8 | 43 |
| Cer(d18:1/16:0) | [M+H]^+^ | 538.60 | 264.40 | 1.8 | 43 |
| Cer(d18:1/18:0) | [M+H]^+^ | 566.70 | 264.40 | 1.7 | 43 |
| Cer(d18:1/20:0) | [M+H]^+^ | 594.60 | 264.40 | 1.7 | 43 |
| Cer(d18:1/20:1) | [M+H]^+^ | 592.60 | 264.40 | 1.7 | 43 |
| Cer(d18:1/22:0) | [M+H]^+^ | 622.70 | 264.40 | 1.7 | 43 |
| Cer(d18:1/22:1) | [M+H]^+^ | 620.70 | 264.40 | 1.7 | 43 |
| Cer(d18:1/24:0) | [M+H]^+^ | 650.80 | 264.40 | 1.8 | 43 |
| Cer(d18:1/24:1) | [M+H]^+^ | 648.80 | 264.40 | 1.8 | 43 |
| Cer(d18:1/26:0) | [M+H]^+^ | 678.90 | 264.40 | 1.8 | 43 |
| Cer(d18:1/26:1) | [M+H]^+^ | 676.90 | 264.40 | 1.7 | 43 |
| SM(d18:1/14:0) | [M+H]^+^ | 675.50 | 184.10 | 9.8 | 44 |
| SM(d18:1/16:0) | [M+H]^+^ | 703.60 | 184.10 | 9.7 | 44 |
| SM(d18:1/18:0) | [M+H]^+^ | 731.60 | 184.10 | 9.6 | 44 |
| SM(d18:1/18:1) | [M+H]^+^ | 729.60 | 184.10 | 9.7 | 44 |
| SM(d18:1/20:0) | [M+H]^+^ | 759.60 | 184.10 | 9.5 | 44 |
| SM(d18:1/20:1) | [M+H]^+^ | 757.60 | 184.10 | 9.5 | 44 |
| SM(d18:1/22:0) | [M+H]^+^ | 787.70 | 184.10 | 9.4 | 44 |
| SM(d18:1/22:1) | [M+H]^+^ | 785.70 | 184.10 | 9.4 | 44 |
| SM(d18:1/24:0) | [M+H]^+^ | 815.70 | 184.10 | 9.3 | 44 |
| SM(d18:1/24:1) | [M+H]^+^ | 813.70 | 184.10 | 9.4 | 44 |
| SM(d18:1/26:1) | [M+H]^+^ | 841.70 | 184.10 | 9.2 | 44 |
| LPE(14:0) | [M-H]- | 424.24 | 283.24 | 8.7 | -50 |
| LPE(16:0) | [M-H]- | 452.27 | 311.27 | 8.7 | -50 |
| LPE(16:1) | [M-H]- | 450.26 | 309.26 | 8.7 | -50 |
| LPE(18:0) | [M-H]- | 480.31 | 339.31 | 8.5 | -50 |
| LPE(18:1) | [M-H]- | 478.29 | 337.29 | 8.5 | -50 |
| LPE(18:2) | [M-H]- | 476.27 | 335.27 | 8.6 | -50 |
| LPE(18:3) | [M-H]- | 474.26 | 333.26 | 8.7 | -50 |
| LPE(20:0) | [M-H]- | 508.34 | 367.34 | 8.4 | -50 |
| LPE(20:1) | [M-H]- | 506.32 | 365.32 | 8.4 | -50 |
| LPE(20:2) | [M-H]- | 504.31 | 363.31 | 8.5 | -50 |
| LPE(20:3) | [M-H]- | 502.29 | 361.29 | 8.0 | -50 |
| LPE(20:4) | [M-H]- | 500.27 | 359.27 | 8.0 | -50 |
| LPE(20:5) | [M-H]- | 498.26 | 357.26 | 8.1 | -50 |
| LPE(22:4) | [M-H]- | 528.31 | 387.31 | 8.4 | -50 |
| LPE(22:5) | [M-H]- | 526.29 | 385.29 | 8.4 | -50 |
| LPE(22:6) | [M-H]- | 524.27 | 383.27 | 8.4 | -50 |
| LPC(14:0) | [M+OAc]^-^ | 526.31 | 227.20 | 10.8 | -50 |
| LPC(16:0) | [M+OAc]^-^ | 554.34 | 255.23 | 10.8 | -50 |
| LPC(16:1) | [M+OAc]^-^ | 552.33 | 253.21 | 10.8 | -50 |
| LPC(18:0) | [M+OAc]^-^ | 582.37 | 283.26 | 10.6 | -50 |
| LPC(18:1) | [M+OAc]^-^ | 580.36 | 281.24 | 10.6 | -50 |
| LPC(18:2) | [M+OAc]^-^ | 578.34 | 279.23 | 10.7 | -50 |
| LPC(18:3) | [M+OAc]^-^ | 576.33 | 277.21 | 10.8 | -50 |
| LPC(20:0) | [M+OAc]^-^ | 610.40 | 311.30 | 10.4 | -50 |
| LPC(20:1) | [M+OAc]^-^ | 608.39 | 309.28 | 10.4 | -50 |
| LPC(20:2) | [M+OAc]^-^ | 606.37 | 307.26 | 10.5 | -50 |
| LPC(20:3) | [M+OAc]^-^ | 604.36 | 305.24 | 10.6 | -50 |
| LPC(20:4) | [M+OAc]^-^ | 602.34 | 303.23 | 10.5 | -50 |
| LPC(20:5) | [M+OAc]^-^ | 600.33 | 301.21 | 10.6 | -50 |
| LPC(22:0) | [M+OAc]^-^ | 638.44 | 339.32 | 10.2 | -50 |
| LPC(22:4) | [M+OAc]^-^ | 630.36 | 331.26 | 10.4 | -50 |
| LPC(22:5) | [M+OAc]^-^ | 628.36 | 329.24 | 10.5 | -50 |
| LPC(22:6) | [M+OAc]^-^ | 626.34 | 327.23 | 10.5 | -50 |
| PC(16:0/14:0) | [M+OAc]^-^ | 764.54 | 227.20 | 7.8 | -51 |
| PC(16:0/16:1) | [M+OAc]^-^ | 790.56 | 253.22 | 7.9 | -51 |
| PC(16:0/18:0) | [M+OAc]^-^ | 820.60 | 283.26 | 7.8 | -51 |
| PC(16:0/18:1) | [M+OAc]^-^ | 818.59 | 281.25 | 7.8 | -51 |
| PC(16:0/20:2) | [M+OAc]^-^ | 844.60 | 307.26 | 7.8 | -51 |
| PC(16:0/20:5) | [M+OAc]^-^ | 838.56 | 301.22 | 7.7 | -51 |
| PC(16:0/22:4) | [M+OAc]^-^ | 868.60 | 331.26 | 7.7 | -51 |
| PC(16:1/18:1) | [M+OAc]^-^ | 816.57 | 281.25 | 7.6 | -51 |
| PC(16:1/18:2) | [M+OAc]^-^ | 814.56 | 253.22 | 7.6 | -51 |
| PC(18:0/14:0) | [M+OAc]^-^ | 792.57 | 227.20 | 7.7 | -51 |
| PC(18:0/18:0) | [M+OAc]^-^ | 848.63 | 283.26 | 7.7 | -51 |
| PC(18:0/20:0) | [M+OAc]^-^ | 876.67 | 283.26 | 7.6 | -51 |
| PC(18:0/20:1) | [M+OAc]^-^ | 874.65 | 309.28 | 7.6 | -51 |
| PC(18:0/20:2) | [M+OAc]^-^ | 872.64 | 307.26 | 7.7 | -51 |
| PC(18:0/20:3) | [M+OAc]^-^ | 870.62 | 305.25 | 7.5 | -51 |
| PC(18:0/20:5) | [M+OAc]^-^ | 866.59 | 301.22 | 7.5 | -51 |
| PC(18:0/22:4) | [M+OAc]^-^ | 896.64 | 331.26 | 7.5 | -51 |
| PC(18:1/18:2) | [M+OAc]^-^ | 842.59 | 279.23 | 7.6 | -51 |
| PC(18:1/18:3) | [M+OAc]^-^ | 840.58 | 277.22 | 7.8 | -51 |
| PC(18:1/20:5) | [M+OAc]^-^ | 864.58 | 301.22 | 7.6 | -51 |
| PC(18:1/22:4) | [M+OAc]^-^ | 894.62 | 331.26 | 7.5 | -51 |
| PC(18:1/22:5) | [M+OAc]^-^ | 892.61 | 329.25 | 7.4 | -51 |
| PC(18:2/20:5) | [M+OAc]^-^ | 862.56 | 301.22 | 7.6 | -51 |
| PC(18:2/22:5) | [M+OAc]^-^ | 890.59 | 329.25 | 7.4 | -51 |
| PC(18:2/22:6) | [M+OAc]^-^ | 888.58 | 327.23 | 7.4 | -51 |
| PE(16:0/14:0) | [M-H]- | 662.48 | 255.23 | 6.1 | -51 |
| PE(16:0/16:0) | [M-H]- | 690.51 | 255.23 | 6.0 | -51 |
| PE(16:0/16:1) | [M-H]- | 688.49 | 253.22 | 6.1 | -51 |
| PE(16:0/18:1) | [M-H]- | 716.52 | 281.25 | 6.0 | -51 |
| PE(16:0/18:2) | [M-H]- | 714.51 | 279.23 | 6.0 | -51 |
| PE(16:0/20:3) | [M-H]- | 740.52 | 305.25 | 5.9 | -51 |
| PE(18:0/16:0) | [M-H]- | 718.54 | 283.26 | 6.0 | -51 |
| PE(18:0/18:0) | [M-H]- | 746.57 | 283.26 | 5.6 | -51 |
| PE(18:0/18:1) | [M-H]- | 744.56 | 281.25 | 5.7 | -51 |
| PE(18:0/18:2) | [M-H]- | 742.54 | 279.23 | 5.9 | -51 |
| PE(18:0/20:1) | [M-H]- | 772.59 | 309.28 | 5.9 | -51 |
| PE(18:0/20:3) | [M-H]- | 768.56 | 305.25 | 5.8 | -51 |
| PE(18:0/20:5) | [M-H]- | 764.52 | 301.22 | 5.8 | -51 |
| PE(18:0/22:4) | [M-H]- | 794.57 | 331.26 | 5.8 | -51 |
| PE(18:1/20:1) | [M-H]- | 770.57 | 309.28 | 5.8 | -51 |
| PE(18:1/22:4) | [M-H]- | 792.56 | 331.26 | 5.8 | -51 |
| PE(18:1/22:6) | [M-H]- | 788.52 | 327.23 | 5.7 | -51 |
| PE(18:2/16:1) | [M-H]- | 712.49 | 279.23 | 6.0 | -51 |

RT, retention time; CE, collision energy; SM, sphingomyelin; Cer, ceramides, LPE, lysophosphatidylethanolamines; LPC, lysophosphatidylcholines; PC, phosphatidylcholines; PE, phosphatidylethanolamines.
